# Synthesis of 2-(4,6-Dimethoxy-1,3,5-triazin-2-yloxyimino) Derivatives: Application in Solution Peptide Synthesis

**DOI:** 10.3390/molecules15129403

**Published:** 2010-12-20

**Authors:** Tarfah I. Al-Warhi, Hassan M.A. AL-Hazimi, Ayman El-Faham, Fernando Albericio

**Affiliations:** 1 Women Students-Medical Studies and Sciences Sections, Chemistry Department, College of Science, King Saud University, P.O. Box 22452, Riyadh 11495, Saudi Arabia; 2 Chemistry Department, College of Science, King Saud University, P.O. Box 2455, Riyadh 11451, Saudi Arabia; 3 Chemistry Department, Faculty of Science, Alexandria University, 426 Ibrahimia, 21321 Alexandria, Egypt; 4 Institute for Research in Biomedicine, Barcelona Science Park, Baldiri Reixac 10, Barcelona 08028, Spain; 5 CIBER-BBN, Networking Centre on Bioengineering, Biomaterials and Nanomedicine, Barcelona Science Park, Baldiri Reixac 10, Barcelona 08028, Spain; 6 Department of Organic Chemistry, University of Barcelona, Martí i Franqués 1-11, Barcelona 08028, Spain

**Keywords:** coupling reagents, triazinyloximino derivatives, solution phase peptide synthesis, uronium salt, racemization

## Abstract

A new class of 1,3,5-triazinyloxyimino derivatives were prepared, characterized and tested for reactivity in solution peptide synthesis. The new triazinyloxyimino derivatives failed to activate the carboxyl group during formation of peptide bonds, but gave the corresponding *N*-triazinyl amino acid derivatives as a major product. The oxyma (ethyl 2-cyano-2-(hydroxyimino)acetate) uronium salt was superior to other uronium salts in terms of racemization, while 2-chloro-4,6-dimethoxy-1,3,5-triazine (CDMT, **9**) gave the best results.

## 1. Introduction

**Figure 1:**
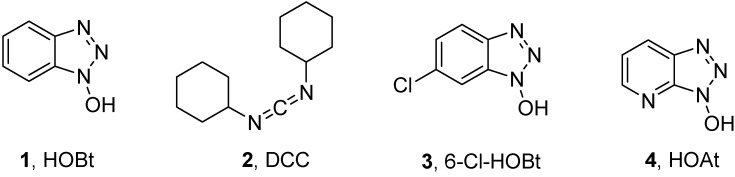
Structure of additives and DCC.

For many years, 1-hydroxybenzotriazole (HOBt, **1**, Figure 1)has been used as a coupling reagent in peptide synthesis, especially in combination with dicyclohexylcarbodiimide (DCC, **2**, Figure 1) [1,2,3,4]. Later, other benzotriazole derivatives such as 6-chloro-1-hydroxybenzotriazole (6-Cl-HOBt, **3**, Figure 1) and 1-hydroxy-7-azabenzotriazole (HOAt, **4**, Figure 1) [5,6] have been introduced and shown more efficient that the parent (HOBt, **1**).

**Figure 2:**
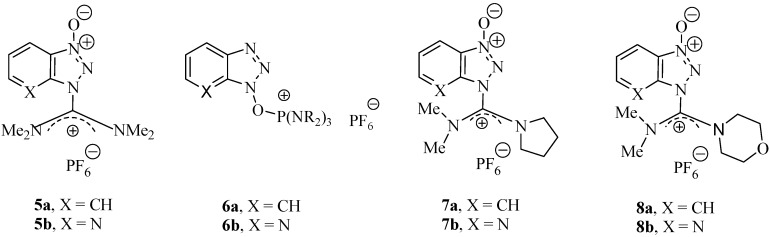
Structure of uronium/iminium salts.

During the last decade, iminium/uronium and phosphonium salts of 1-hydroxybenzotriazole and 1-hydroxy-7-azabenzotriazole, **5 **and **6 **(Figure 2)have shown the supremacy of these kind of derivatives over carbodiimides in the presence of an additive [5,6,7,8,9,10,11,12,13,14,15,16,17,18,19,20,21,22,23,24,25,26,27,28,29,30,31,32,33].

Despite their widespread use, these reagents are not without ptoblems. The explosive properties of 1-hydroxybenzotriazoles are often not referenced in literature. Sometime, Material Safety Data Sheets warn that 1-hydroxybenzotriazoles may be unstable, with a relatively high sensitivity to friction and sparks, but in most cases, there is no mention of how sensitive such substances are to heating under confinement and no warning is given with respect to their ability to propagate a deflagration or a detonation.

More recently a new class of coupling reagents such as **7** and **8** (Figure 2) based on changes to the carbon skeleton structure [34,35,36] gave more marked increases in coupling efficiency, as well as reduced racemization levels during the coupling step. Particularly noteworthy are the morpholino derivatives **8**, because the oxygen in the carbon skeleton increases the solubility as well as the reactivity, which reduces the racemization during the coupling step [34,35,36].

Although 2-chloro-4,6-dimethoxy-1,3,5-triazine (CDMT, **9**) has been successfully applied for the preparation of peptides, the mechanism of its participation in coupling reactions remains unknown [37,38]. Furthermore, in order to be successfully stored the reagent itself must be of high purity, the formation of gaseous products may generate a rapid pressure increase in the container, and accordingly, precautions should be taken to avoid the serious risk of blowout of toxic gases.

**Figure 3:**
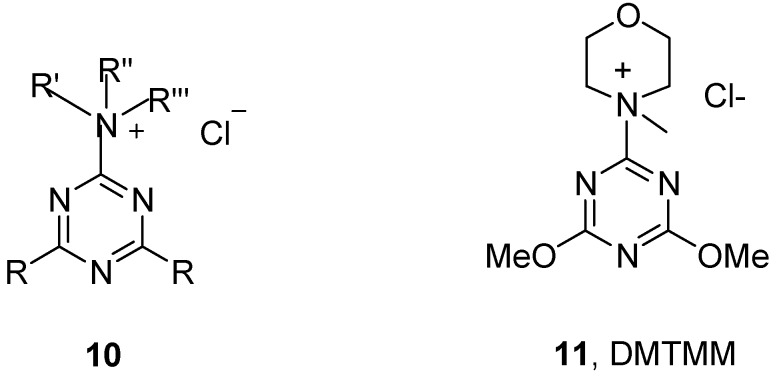
Structure of triazinylammonium salts.

Successful activation of carboxylic acids by means of CDMT (**9**) confirmed the feasibility of a multistep process proceeding via triazinylammonium salts such as **10** and (DMTMM, **11**) (Figure 3)formed *in situ* in the presence of the appropriate amine [37,38].

The related triazine, 4-(4,6-dimethoxy-1,3,5-triazin-2-yl)-4-methylmorpholinium chloride (DMTMM, **11**), formed by a simple reaction of CDMT (**9**) with *N*-methylmorpholine (NMM), has been found applications in amidation [38,39,40,41,42], esterification [42], glycosidation [43,44] and phosphonylation [45] methodology. The present work presents the synthesis and application of a new family of 1,3,5-triazine derivatives and their comparison with CDMT (**9**) as well as the uronium based type coupling reagents [46].

## 2. Results and Discussion

**Scheme 1:**
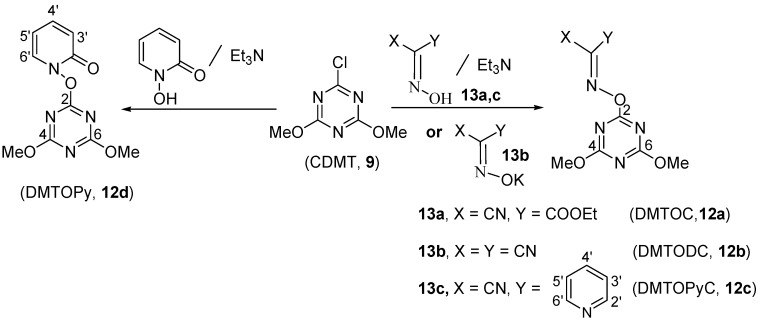
Synthesis of triazinyloxyimino derivatives.

The new triazinyloxyimino derivatives **12a-c** were prepared from the reaction of the oxime derivatives **13a-c** with CDMT (**9**) in the presence of triethylamine. The potassium salts were used in case of the synthesis of malononitrile and diethylmalonate derivatives (Scheme 1). 

**Scheme 2:**
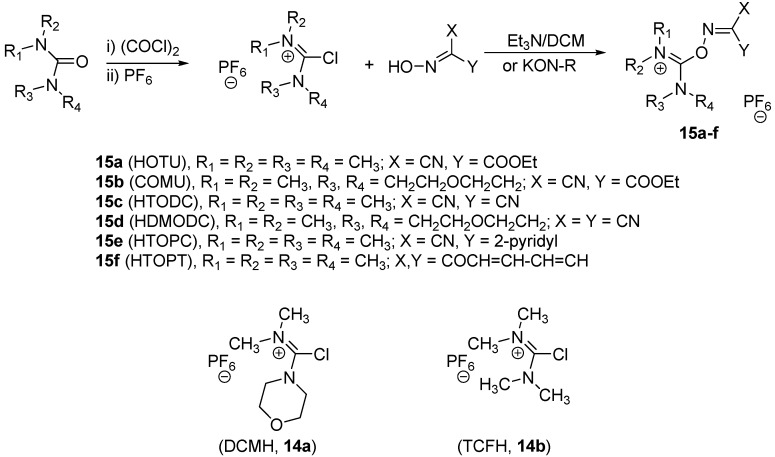
Synthesis of uronium salts.

The pyridinyl derivative was prepared using the same conditions to afford **12d**. All the products **12a-d** have been identified on the basis of their spectroscopic data (see Experimental). In order to have a good comparison to the triazinyloxyimino coupling reagents, some of the related uronium-type coupling reagents were prepared according to the reported method [36] as shown in Scheme 2. 

**Figure 4:**
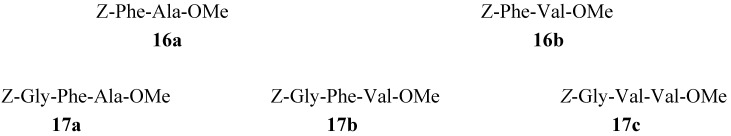
Model of peptide used in racemization studies.

The uronium salt coupling reagents were prepared by reaction of the chloro salts DCMH **14a** or TCFH **14b **with the oxime derivatives in the presence of triethylamine in DCM or with the potassium salt of the oxime derivatives in acetonitrile to give the oxyimino uronium base coupling reagents **15a-f** (Scheme 2). To investigate the retention of configuration induced for the new coupling reagents, several previously studied model peptide system **16a,b** and **17a-c** (Figure 4) were examined [36]. These models involve stepwise coupling and (2+1) segment coupling as well.

Results comparing the coupling of *Z*-Phe-OH to H-Ala-OMe.HCl (**18a**) with different coupling agents to afford the *Z*-Phe-Ala-OMe (**16a**) are indicated in Table 1. All the uronium type coupling reagents (HOTU, **15a**), (HTOPC, **15e**), and (HTOPT, **15f**), used gave less than 1% racemization according to the NMR data but CDMT (**9**) gave about 15% of the racemized product (Table 1).

**Table 1:** Yield (%) and Racemization (%) of *Z*-Phe-Ala-OMe (**16a**) using the Uronium Salts **15a,e,f **and CDMT (**9**).

Coupling Reagent	Yield %	DL %
(HOTU, **15a**)	100	<1
(HTOPC, **15e**)	54	<1
(HTOPT, **15f**)	66	<1
(CDMT, **9**)	55	15

CH_3_ DL (d) at δ = 1.20 ppm; CH_3_ LL (d) at δ = 1.32 ppm.

**Scheme 3:**
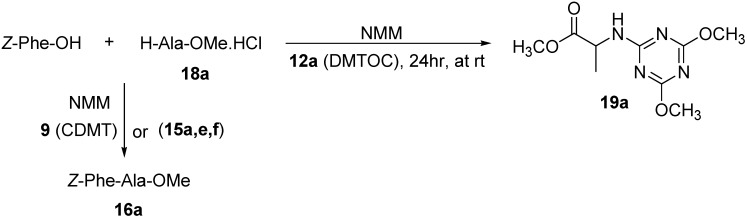
Coupling of Z-Phe-OH with H-Ala-OMe.HCl (**18a**) using DMTOC (**12a**) and CDMT (**9**).

The other triazinyloxyimino derivative (DMTOC, **12a**) failed to give the expected product *Z*-Phe-Ala-OMe (**16a**), due to the low activation for the carboxylic group and fast attack by the *N*-terminal of the amino acid which led to formation of the *N*-triazinyl amino acid derivative methyl 2-(4,6-dimethoxy-1,3,5-triazin-2-ylamino)propanoate (**19a**) in 77-85% yield, as confirmed by NMR data (Scheme 3).

**Scheme 4:**
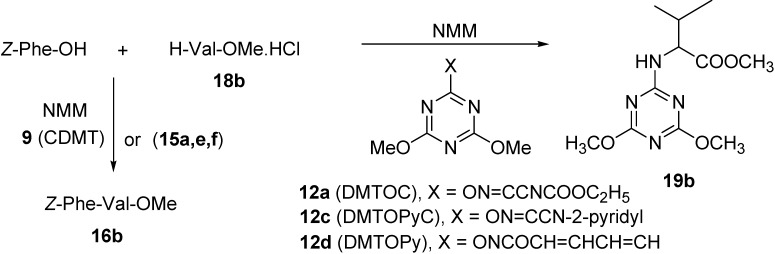
Coupling of Z-Phe-OH with H-Val-OMe.HCl (**18b**) using triazinyloxyimino derivatives and CDMT (**9**).

Another example, the coupling of *Z*-Phe-OH to H-Val-OMe.HCl (**18b**) to afford the dipeptide *Z*-Phe-Val-OMe (**16b**) showed the same behavior for the uronium salts and triazinyloxyimino derivatives (Scheme 4, Table 2).

**Table 2:** Yield (%) and Racemization (%) of *Z*-Phe-Val-OMe (**16b**) using the Uronium Salts **15a,e,f** and CDMT (**9**).

Coupling Reagent	Yield %	DL %
(HOTU, **15a**)	83	<1
(HTOPC, **15e**)	89	<1
(HTOPT, **15f**)	68	<1
(CDMT, **9**)	69	2.0

CH_3_ DL (dd) at δ = 0.66 ppm; CH_3_ LL (dd) at δ = 0.82 ppm.

**Scheme 5:**
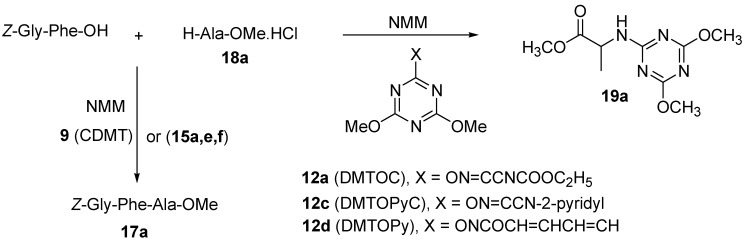
Coupling of Z-Gly-Phe-OH with H-Ala-OMe.HCl (**18a**) using triazinyloxyimino derivatives and CDMT (**9**).

The desired dipeptide product was not formed in case of the triazinyloxyimino derivatives (DMTOC, **12a**), (DMTOPyC, **12c**), and (DMTOPy, **12d**) but rather the side product methyl 2-(4,6-dimethoxy-1,3,5-triazin-2-ylamino)-3-methylbutanoate (**19b**), due to the reaction of amino acid ester with the coupling reagents as indicated by NMR. For the rather non-sensitive case of segment coupling of *Z*-Gly-Phe-OH to H-Ala-OMe.HCl (**18a**), which leads to the tripeptide *Z*-Gly-Phe-Ala-OMe (**17a**), the Oxyma (ethyl 2-cyano-2-(hydroxyimino)acetate) derivatives gave the best results compared with others as observed from the NMR data (Scheme 5, Table 3).

**Table 3:** Yield (%) and Racemization of (%)*Z*-Gly-Phe-Ala-OMe (**17a**) using the Uronium Salts **15a,e,f** and CDMT (**9**).

Coupling Reagent	Yield %	DL %
(HOTU, **15a**)	63	<1
(HTOPC, **15e**)	19	19.0
(HTOPT, **15f**)	76	12.8
(CDMT, **9**)	gummy	30

CH_3_ DL (d) at δ = 1.19 ppm; CH_3_ LL (d) at δ = 1.32 ppm.

The rest of the triazinyloxyimino derivatives (DMTOC, **12a**), (DMTOPyC, **12c**), and (DMTOPy, **12d**) did not afford the desired product, giving the side product methyl 2-(4,6-dimethoxy-1,3,5-triazin-2-ylamino)propanoate (**19a**) (Scheme 5) as a major product, as revealed from its NMR spectral data.

In the more sensitive cases of the formation of *Z*-Gly-Phe-Val-OMe (**17b**) and *Z*-Gly-Val-Val-OMe(**17c**) the same results were obtained. The best results obtained were when the Oxyma derivative (HOTU, **15a**) was using as a coupling reagent, while CDMT (**9**) gave the highest racemization level and the triazine coupling reagents failed in the formation of the target product, giving the same side product **19b**, which was obtained from the coupling of *Z*-Phe-OH with H-Val-OMe.HCl (**18b**) as observed from the HPLC and NMR data (Table 4 and Table 5).

**Table 4:** Yield (%) and Racemization of (%) *Z*-Gly-Phe-Val-OMe (**17b**) using the Uronium Salts **15a,e,f** and CDMT (**9**).*

Coupling Reagent	Yield %	DL %
(HOTU, **15a**)	80	< 0.1
(HTOPC, **15e**)	81	26.19
(HTOPT, **15f**)	78	6.15
(CDMT, **9**)	63	50.0

* The racemization percent was detected by reverse phase HPLC, using a SunFire C_18_ column (Waters) (5 μm, 4.6 × 150 mm), linear gradient 10-90 0.036% TFA in CH_3_CN/0.045% TFA in H_2_O over 8 min *t_R_* LL is 7.43 min, *t_R_* DL is 7.61 min as indicated from and authentic samples.

**Table 5:** Yield (%) and Racemization (%) of *Z*-Gly-Val-Val-OMe (**17c**) using the Uronium Salts **15a,e,f** and CDMT (**9**).*

Coupling Reagent	Yield %	DL %
(HOTU, **15a**)	81	< 0.1
(HTOPC, **15e**)	≈100	17.5
(HTOPT, **15f**)	95	6.66
(CDMT, **9**)	60	47.3

* The percent racemization was detected by reverse phase HPLC, using a SunFire C_18_ column (Waters) (5 μm, 4.6 × 150 mm), linear gradient 10-90 0.036% TFA in CH_3_CN/0.045% TFA in H_2_O over 8 min *t_R_* LL is 6.90 min, *t_R_* DL is 7.07 min as indicated by authentic samples.

## 3. Experimental

### 3.1. General

All chemicals were used without further purification. Solvents are redistilled before use. *N*-protected amino acids and esters were purchased from Novabiochem. Melting points are uncorrected and were determined on a Gallenkamp hot stage. A Perkin Elmer Spectrum 1000 FT-IR Spectrometer was used for recording infrared (IR) spectra of the prepared compounds as KBr pellets or in spectroscopic grade dichloromethane. ^1^H- and ^13^C-NMR spectra of compounds were run on JEOL 400 MHz NMR spectrometer, in CD_3_COCD_3_, CDCl_3_ or DMSO-d_6_ at room temperature using TMS as internal standard. The instruments are located at King Saud University, College of Science, Chemistry Department. For analytical separations, characterization and determination of racemization, a reverse-phase Waters 2695 HPLC separation module was used (Barcelona Science Park, University of Barcelona, Spain) equipped with a SunFire C_18_ column (Waters, 5 μm, 4.6 × 150 mm), 10-90% linear gradient of 0.036% TFA in CH_3_CN/0.045% TFA in H_2_O and coupled to a Waters 2998 PDA-UV detector. Chromatograms were processed with Empower software. In the detection of the percent racemization as indicated by authentic samples. Peptide mass was detected by means of an HPLC-PDA system as described above, coupled to a Water Micromass ZQ mass detector, using the MassLynx 4.1 software. Elemental analyses were performed on a Perkin-Elmer 2400 elemental analyzer, and the values found were within ±0.3% of the theoretical values. Oxime derivatives **13a-c** were prepared following the literature procedures [47,48].

### 3.2. General Synthesis of Triazine Coupling Reagents **12a-d** from Oxime Derivatives

2-Chloro-4,6-dimethoxy-1,3,5-triazine (**9**, 20 mmol) was added to a solution of oxime (20 mmol) and triethylamine (20 mmol) in dichloromethane (DCM, 200 mL) at 0 ºC. The reaction mixture was stirred at the same temperature for 1 h and then at room temperature for 5-8 h. DCM (400 mL) was added, and then the reaction mixture was washed twice with saturated aqueous NaCl (200 mL each). Finally, the organic solvent was dried with anhydrous Na_2_SO_4_, filtered, and the solvent was removed under reduced pressure. The crude product was recrystallized from DCM/hexane.

*Ethyl-2-cyano-2-(4,6-dimethoxy-1,3,5-triazin-2-yloxyimino)acetate* (DMTOC, **12a**). White powder, yield 4.87 g (86%), m.p. 110 ºC; IR (KBr): 2986.68 (aliph. CH), 2363.98, 2344.92 (C≡N), 1758.25 (C=O, ester), 1608.79 (C=N), 1286.58 (N-O), 1084.42 (C-O-C) cm^-1^; ^1^H-NMR (CDCl_3_): δ 1.44 (t, *J* = 7.2 Hz, 3H, CH_3_), 4.11 (s, 6H, 2 OCH_3_), 4.50 (q, *J* = 7.2 Hz, 2H, CH_2_) ppm; ^13^C-NMR (CDCl_3_): δ 14.19 (CH_3_), 56.36 (2 OCH_3_), 64.77 (CH_2_), 106.95 (C≡N), 131.58 (C=N), 156.88 (C=O), 172.96 (C-2), 174.18 (C-4,6) ppm; Elemental analysis, calculated for C_10_H_11_N_5_O_5_ (281.22): C, 42.71; H, 3.94; N, 24.90. Found: C, 42.46; H, 3.82; N, 24.66.

*(4,6-Dimethoxy-1,3,5-triazin-2-yloxy)carbonimidoyl dicyanide* (DMTODC, **12b**). Snow white powder, yield 3.11 g (66%), m.p. 74 ºC; IR (KBr): 2948.10 (aliph. CH), 2363.80, 2344.87 (C≡N), 1558.96 (C=N), 1467.87 (Ar. C=N), 1361.80 (Ar. C-N), 1222.97 (N-O), 1042.73 (C-O-C) cm^-1^; ^1^H-NMR (CDCl_3_): δ 3.37 (s, 6H, 2 OCH_3_) ppm; ^13^C-NMR (CDCl_3_): δ 56.58 (2 OCH_3_), 105.11, 108.19 (2 C≡N), 114.80 (C=N), 172.34 (C-2), 174.21 (C-4,6) ppm; Elemental analysis, calculated for C_8_H_6_N_6_O_3_ (234.17): C, 41.03; H, 2.58; N, 35.89. Found: C, 41.16; H, 2.49; N, 35.68.

*N-(4,6-Dimethoxy-1,3,5-triazin-2-yloxy)picolinimidoyl cyanide* (DMTOPyC, **12c**). Pink cotton solid, yield 6.29 g (≈100%) from (10 mmol of starting material), m.p. 198 ºC; IR (KBr): 3468.01 (Ar. C-H), 2924.86 (aliph. CH), 2364.64, 2344.88 (C≡N), 1606.27 (C=N, C=C), 1560.18 (asym. C-O-C), 1361.76 (sym. C-O-C) cm^-1^; ^1^H-NMR (CDCl_3_): δ 4.13 (s, 6H, 2 OCH_3_), 7.52 (m, 1H, H-5'), 7.86 (dt, 1H, H-4'), 8.22 (d, *J* = 7.32 Hz, 1H, H-3'), 8.82 (d, *J* = 4.4 Hz, 1H, H-6') ppm; ^13^C-NMR (CDCl_3_): δ 56.11 (2 OCH_3_), 108.21 (C≡N), 122.26 (C-3'), 126.74 (C-5'), 137.21 (C-4'), 139.89 (C-6'), 147.42 (C-2'), 150.43 (C=N), 173.26 (C-2), 174.04 (C-4,6) ppm; Elemental analysis, calculated for C_12_H_10_N_6_O_3_ (286.25): C, 50.35; H, 3.52; N, 29.36. Found: C, 50.59; H, 3.42; N, 29.29.

*1-(4,6-Dimethoxy-1,3,5-triazin-2-yloxy)pyridin-2(1H)-one* (DMTOPy, **12d**). The product was obtained as a light tan powder, yield 4.28 g (85%), m.p. (104-106 ºC); IR (KBr): 3091.57 (Ar. C-H), 2953.80 (aliph. CH), 1678.35 (C=O), 1608.62 (C=N, C=C), 1558.30 (N-O), 1369.99, 1349.16 (Ar. C-N), 1280.22 (asym. C-O-C), 1084.36 (sym. C-O-C) cm^-1^; ^1^H-NMR (CDCl_3_): δ 3.96 (s, 6H, 2 OCH_3_), 6.25 (dt, 1H, H-5'), 6.75 (dd, 1H, H-3'), 7.42 (1H, H-4'), 7.53 (dd, H-6') ppm; ^13^C-NMR (CDCl_3_): δ 56.10 (2 OCH_3_), 105.39 (C-5'), 123.27 (C-3'), 135.45 (C-6'), 139.79 (C-4'), 157.23 (C=O), 173.96 (C-2), 174.11 (C-4,6) ppm; Elemental analysis, calculated for C_10_H_10_N_4_O_4_ (250.21): C, 48.00; H, 4.03; N, 22.39. Found: C, 48.22; H, 4.09; N, 22.45.

### 3.3. General Synthesis of Oximo-Uronium Type Coupling Reagents **15a-f**

To a solution of the oxime potassium salts of **13a-c** (20 mmol) in acetonitrile (ACN) (50 mL) was added to the chloro salts **14a,b** (20 mmol) at 0 ºC. The reaction mixture was stirred at this temperature 30 min and stirred at room temperature for 6 h. Filter and wash with acetonitrile. The solvent was concentrated to small volume (1/4) under reduced pressure, and then dry ether was added to afford the product as a white solid in pure state [46].

*O-[(Cyano(ethoxycarbonyl)methylidene)amino]-1,1,3,3-tetramethyluronium hexafluorophosphate* (HOTU, **15a**). Yield 6.32g (82%) using the potassium salt of the oxime, m.p. (135-137 ºC) (dec). The triethylamine/oxime combinations gave a lower yield (69%); IR (KBr): 3003.32 (N-CH_3_), 2964.16 (aliph. CH), 2345.36 (C≡N, =N+), 1771.32 (C=O), 1702.40 (C=N), 1266.93 (N-O) cm^-1^; ^1^H-NMR (acetone-d_6_): δ 1.37 (t, 3H, CH_3_), 3.37 (s, 12H, 4 CH_3_), 4.82 (q, 2H, CH_2_) ppm; ^13^C-NMR (acetone-d_6_): δ 13.46 (CH_3_), 40.71 (4 CH_3_), 64.56 (CH_2_), 106.78 (C≡N), 135.09 (C=N), 156.11 (C=O), 161.43 (C+) ppm; Elemental Analysis, calculated for C_10_H_17_F_6_N_4_O_3_P (386.23): C, 31.10; H, 4.44; N, 14.51. Found: C, 31.33; H, 4.40; N, 14.36.

*1-[(1-(Cyano-2-ethoxy-2-oxoethylideneaminooxy)dimethylaminomorpholinomethylene)]methanamin- ium hexafluorophosphate* (COMU, **15b**). Yield 7.6g (89%), m.p. (143-144 ºC); ^1^H-NMR (acetone-d_6_): δ 1.38 (t, 3H, CH_3_), 3.41 (s, 6H, 2 CH_3_), 3.82 (t, 4H, 2 CH_2_), 3.89 (t, 4H, 2 CH_2_), 4.48 (q, 2H, CH_2_) ppm; ^13^C-NMR (acetone-d_6_): δ 13.48 (CH_3_), 40.70 (2 CH_3_), 49.94 (2 CH_2_), 64.59 (2 CH_2_), 66.04 (CH_2_), 106.76 (C≡N), 135.03 (C=N), 156.14 (C=O), 160.61 (C+) ppm; Elemental Analysis, calculated for C_12_H_19_F_6_N_4_O_4_P (428.27): C, 33.65; H, 4.47; N, 13.08. Found: C, 33.92; H, 4.41; N, 13.24.

*O-[(Dicyanomethylidene)amino]-1,1,3,3-tetramethyluronium hexafluorophosphate* (HTODC, **15c**). Yield 5.0 g (74%), m.p. (180-181 ºC) (dec); ^1^H-NMR (acetone-d_6_): δ 3.27 (s, 12H, 4 CH_3_) ppm; ^13^C-NMR (acetone-d_6_): δ 40.80 (4 CH_3_), 105.10 (C≡N), 108.21 (C≡N), 119.65 (C=N), 160.67 (C+) ppm; Elemental Analysis, calculated for C_8_H_12_F_6_N_5_OP (339.18): C, 28.33; H, 3.57; N, 20.65. Found: C, 28.56; H, 3.64; N, 20.80.

*1-[(1-(Dicyanomethyleneaminooxy)dimethylaminomorpholinomethylene)]methanaminium hexafluoro- phosphate* (HDMODC, **15d**). Yield 5.7 g (75%), m.p. (118-119 ºC); ^1^H-NMR (acetone-d_6_): δ 3.41 (s, 6H, 2 CH_3_), 3.87-3.88 (m, 8H, 4 CH_2_) ppm; ^13^C-NMR (acetone-d_6_): δ 40.97 (2 CH_3_), 49.93 (2 CH_2_), 65.92 (2 CH_2_), 105.13 (C≡N), 108.15 (C≡N), 119.84 (C=N), 159.77 (C+) ppm; Elemental Analysis, calculated for C_10_H_14_F_6_N_5_O_2_P (381.21): C, 31.51; H, 3.70; N, 18.37. Found: C, 31.77; H, 3.56; N, 18.52.

*N-[(Cyano(pyridine-2-yl)methyleneaminooxy)(dimethylamino)methylene]-N-methylmethanaminium hexafluorophosphate* (HTOPC, **15e**). Yield 6.2 g (83%), m.p. (169-171 ºC); IR (KBr): 3069.24 (N-CH_3_), 2966.06 (aliph. CH), 2345.51 (C≡N, =N+), 1692.23 (C=N, C=C), 1273.86 (N-O) cm^-1^; ^1^H-NMR (DMSO-d_6_): δ 3.21 (s, 12H, 4 CH_3_), 7.74 (t, 1H, H-5), 8.07 (t, 1H, H-4), 8.14 (d, *J* = 8.08 Hz, 1H, H-3), 8.85(d, *J* = 5.12 Hz, 1H, H-6) ppm; ^13^C-NMR (DMSO-d_6_): δ 40.27 (4 CH_3_), 108.54 (C≡N), 122.74 (C-3), 128.23 (C-5), 138.62 (C-4), 142.07 (C-2), 146.70 (C-6), 150.88 (C=N), 161.17 (C+) ppm; Elemental Analysis, calculated for C_12_H_16_F_6_N_5_OP (391.21): C, 36.84; H, 4.12; N, 17.90. Found: C, 37.06; H, 4.22; N, 18.12.

*O-(2-Pyridone)-1,1,3,3-tetramethyluronium hexafluorophosphate* (HTOPT, **15f**). Yield 6.4 g (82%), m.p. (166-176 ºC); IR (KBr): 3096.77 (N-CH_3_), 2958.98 (aliph. CH), 2345.56 (=N+), 1709.64 (C=O), 1672.42 (C=C, C=N), 1272.58 (N-O) cm^-1^; ^1^H-NMR (DMSO-d_6_): δ 3.11 (s, 12H, 4 CH_3_), 6.52 (t, *J* = 7.36 Hz, 1H, H-5), 6.78 (d, *J* = 8.8, 1H, H-3), 7.65 (t, *J* = 7.32 Hz, 1H, H-4), 8.42 (d, *J* = 7.32 Hz, 1H, H-6) ppm; ^13^C-NMR (DMSO-d_6_): δ 40.27 (4 CH_3_), 107.02 (C-5), 121.98 (C-3), 135.90 (C-4), 142.05 (C-6), 156.56 (C=O), 162.12 (C+) ppm; Elemental Analysis, calculated for C_14_H_18_F_6_N_5_O_2_P (391.21), C, 38.81; H, 4.19; N, 16.16. Found: C, 39.03; H, 4.31; N, 16.34.

### 3.4. Synthesis of **16a,b** (1+1)

The coupling reagents **15a,e,f** (0.25 mmol) were added to a mixture of *Z*-Phe-OH (0.25 mmol), the appropriate amino acid **18a,b** (0.25 mmol) and *N*-methylmorpholine (0.75 mmol), in dry acetonitrile (5 mL) at 0 ºC. The reaction mixture was stirred at the same temperature for 1 h and then at room temperature for 2 h. Ethyl acetate (EtOAc, 50 mL) was added and the mixture was subsequently washed with 10% aqueous HCl (v/v), saturated aqueous Na_2_CO_3_ and saturated aqueous NaCl solution (2 × 10 mL each). Finally, the organic solvent was dried with anhydrous Na_2_SO_4_, filtered, and the solvent was removed under reduced pressure. The gummy residue obtained **16a,b** (DL < 1%) were dried under vacuum. Both products were obtaind when the coupling chlorotriazine derivative **9 **was used (15% racemization). Using the triazine coupling reagent **12a,c,d **instead of **15a,e,f** and following the different procedure whereby *Z*-Phe-OH was activated with **12a,c,d** for 1 hour at room temperature and then added to a solution of **18a,b **in the presence of NMM as a base in acetonitrile, the reaction mixture was stirred at the same temperature for 24 h gave compounds **19a,b**.

*Z-Phe-Ala-OMe* (**16a**). IR (KBr): 3303.71 (NH), 2951.65, 2926.12 (aliph. CH), 1743.34 (C=O, ester), 1694.14 (C=O, urethane), 1652.91 (C=O, amide) cm^-1^; ^1^H-NMR (CDCl_3_): δ 1.31 (d, 3H, CH_3_), 3.06 (d, 2H, CH_2_-C), 3.72 (s, 3H, OCH_3_), 4.47-4.51 (m, 2H, 2 CH), 5.07 (s, 2H, CH_2_-O), 5.38 (1H, NH, amide), 6.42 (1H, NH, urethane), 7.25-7.32 (m, 10H, Ar. H) ppm; ^13^C-NMR (CDCl_3_): δ 18.36 (CH_3_), 38.64 (CH_2_-C), 48.23 (CH_2_-O), 52.57 (OCH_3_), 56.10 (CH, Phe), 67.16 (CH, Ala), 129.43 (C=O, amide), 170.43 (C=O, urethane), 172.88 (C=O, ester) ppm.

*Z-Phe-Val-OMe* (**16b**). IR (NaCl/DCM): 3310.29 (NH), 2963.93 (aliph. CH), 1741.07 (C=O, ester), 1704.99 (C=O, urethane), 1661.22 (C=O, amide) cm^-1^; ^1^H-NMR (CDCl_3_): δ 0.80-0.84 (dd, 6H, 2 CH_3_), 2.02-2.12 (m, 1H, CH(CH_3_)_2_), 3.05-3.07 (m, 2H, CH_2_-C), 3.68 (s, 3H, OCH_3_), 4.45-4.46 (m, 2H, 2 CH), 5.08 (s, 2H, CH_2_-O), 5.42 (1H, NH, amide), 6.36 (1H, NH, urethane), 7.24-7.32 (m, 10H, Ar. H) ppm.

### 3.5. General Method for Synthesis of **17a-c** (2+1) Using Different Coupling Reagents

The coupling reagents **15a,e,f** (0.25 mmol) were added to a mixture of *Z*-Gly-Phe-OH (0.25 mmol), **18a,b **(0.25 mmol) and *N*-methylmorpholine (0.75 mmol), in dry ACN (5 mL) at 0 ºC. The reaction mixture was stirred at the same temperature for 1 h and then at room temperature for 24 h. Ethyl acetate (EtOAc, 50 mL) was added and the mixture was subsequently washed with 10% aqueous HCl (v/v), saturated aqueous Na_2_CO_3_ and saturated aqueous NaCl solution (2 × 10 mL each). Finally, the organic solvent was dried with anhydrous Na_2_SO_4_, filtered, and the solvent was removed under reduced pressure. The gummy residue obtained **17a,b** (DL < 1% in the case of using **15a**) was dried under vacuum. Both products were obtained when the coupling chlorotriazine **9 **was used. Using the triazine coupling reagent **12a,c,d **instead of **15a,e,f** and following the different procedure whereby *Z*-Gly-Phe-OH was activated with **12a,c,d** for 1 h at 0 ºC and then added to a solution of **18a,b **in the presence of NMM as a base in acetonitrile, the reaction mixture was stirred at the same temperature for 1 h, then at room temperature for 24 h gave compounds **19a,b**.

*Z-Gly-Phe-Ala-OMe* (**17a**). IR (NaCl/DCM): 3434.81, 3272.55 (NH), 2950.43 (aliph. CH), 1735.95 (C=O, ester), 1718.13 (C=O, urethane), 1685.96, 1647.73 (2 C=O, amide) cm^-1^; ^1^H-NMR (CDCl_3_): δ 1.30 (d, 3H, CH_3_), 3.03 (d, 2H, CH_2_-N), 3.67 (s, 3H, OCH_3_), 3.79-3.85 (m, 2H, CH_2_-C), 4.43-4.46 (m, 1H, CH, Phe), 4.68-4.70 (m, 1H, CH, Ala), 5.08 (s, 2H, CH_2_-O), 5.62, 6.71 (2H, 2 NH, amide), 6.91 (1H, NH, urethane), 7.14-7.32 (m, 10H, Ar. H) ppm.

*Z-Gly-Phe-Val-OMe* (**17b**). IR (NaCl/DCM): 3298.25 (br. NH), 2961.81 (aliph. CH), 1719.76 (br. C=O, ester & urethane), 1654.46 (2 C=O, amide) cm^-1^; ^1^H-NMR (CDCl_3_: δ 0.77-0.82 (dd, 6H, 2 CH_3_), 2.02-2.12 (m, 1H, CH(CH_3_)_2_), 2.90-3.10 (m, 2H, CH_2_-N), 3.62 (s, 3H, OCH_3_), 3.60-3.90 (m, 2H, CH_2_-C), 4.35-4.49 (m, 1H, CH, Gly), 4.75-4.85 (m, 1H, CH, Val), 5.06 (s, 2H, CH_2_-O), 5.85, 6.95 (2H, 2 NH, amide), 7.12-7.20 (m, 1H, NH, urethane), 7.25-7.36 (m, 10H, Ar. H) ppm.

*Z-Gly-Val-Val-OMe* (**17c**). Prepared following the same procedure above for **17a,b** by replacing *Z*-Gly-Phe-OH with *Z*-Gly-Val-OH, and the amino acid **18b** was used. The gummy residue of **17c **obtained when the coupling reagents **15a,e,f** (DL < 0.1% in the case of using **15a**) was dried under vacuum. The product was obtained (47.3% racemization) when the coupling chlorotriazine **9 **was used. Using the triazine coupling reagent **12a,c,d **instead of **15a,e,f** and following the different procedure whereby *Z*-Gly-Val-OH was activated with **12a,c,d** for 1 hour at 0 ºC and then added to a solution of **18b **in the presence of NMM as a base in acetonitrile, the reaction mixture was stirred at room temperature for 24 h to give compound **19b**.

*Z-Gly-Val-Val-OMe* (**17c**). IR (NaCl/DCM): 3411.60, 3303.57 (NH), 2967.43 (aliph. CH), 1733.86 (br. C=O, ester & urethane), 1651.45 (2 C=O, amide) cm^-1^; ^1^H-NMR (CDCl_3_): δ 0.88-0.90 (dd, 12H, 4 CH_3_), 2.06-2.14 (m, 2H, 2 CH(CH_3_)_2_), 3.70 (s, 3H, OCH_3_), 3.90 (2H, CH_2_-N), 4.38 (1H, CH, Gly), 4.48-4.51 (m, 1H, CH, Val), 5.10 (s, 2H, CH_2_-O), 5.68, 6.74 (2H, 2 NH, amide), 6.82 (1H, NH, urethane), 7.25-7.34 (m, 5H, Ar. H) ppm. 

*Methyl-2-(4,6-dimethoxy-1,3,5-triazin-2-ylamino)propanoate* (**19a**). m.p. 98-100 ºC, ^1^H-NMR (CDCl_3_): δ 1.48 (d, 3H, CH_3_), 3.72 (s, 3H, CH_3_ ester), 3.93 (s, 6H, 2 OCH_3_), 4.70 (q, 1H, CH), 5.93 (d, 1H, NH) ppm; Elemental analysis calculated for C_9_H_14_N_4_O_4_: C, 44.63; H, 5.83; N, 23.13. Found: C, 44.91; H, 6.00; N, 23.36.

*Methyl-2-(4,6-dimethoxy-1,3,5-triazin-2-ylamino)-3-methylbutanoate* (**19b**). m.p. 103-105 ºC,^ 1^H-NMR (CDCl_3_): δ 0.96 (t, 6H, 2 CH_3_), 2.21-2.22 (m, 1H, CH), 3.72 (s, 3H, CH_3_ ester), 3.91 (s, 6H, 2 OCH_3_), 4.67 (q, 1H, CH), 5.84 (d, 1H, NH) ppm; Elemental analysis calculated for C_11_H_18_N_4_O_4_: C, 48.88; H, 6.71; N, 20.73. Found: C, 49.12; H, 6.89; N, 21.01.

## 4. Conclusions

In conclusion, herein a new family of 1,3,5-triazinyloxyimino derivatives **12a-d** was compared with uronium salts. The morpholino and tetramethyluronium salts are more reactive than triazinyloxyimino derivatives for activation of the carboxylic group and formation of peptide bonds. The new triazinyloxyimino derivatives **12a-d** failed to give the expected products, due to the low activation for the carboxylic group and fast attack by the *N*-terminal of the amino acid, which led to formation of the corresponding *N*-triazinyl amnio acid derivatives as confirmed by NMR and HPLC. Oxyma (ethyl 2-cyano-2-(hydroxyimino)acetate) uronium salts were confirmed to be superior to other oxime derivatives, HOAt (**4**) and HOBt (**1**), in terms of both coupling yield and retention of configuration. Finally, the new triazinyloxyimino derivatives may be useful in the preparation of biologically active *N*-triazinyl amino acids as well as *N*-triazinyl peptides which is now going to be tested in our laboratory.
